# Advancing a temporal framework for understanding the biology of nonsuicidal self-injury: An expert review

**DOI:** 10.1016/j.neubiorev.2021.08.022

**Published:** 2021-08-24

**Authors:** Michael Kaess, Jill M. Hooley, Bonnie Klimes-Dougan, Julian Koenig, Paul L. Plener, Corinna Reichl, Kealagh Robinson, Christian Schmahl, Maurizio Sicorello, Mindy Westlund Schreiner, Kathryn R. Cullen

**Affiliations:** a University Hospital of Child and Adolescent Psychiatry and Psychotherapy, University of Bern, Bern, Switzerland; b Department of Child and Adolescent Psychiatry, Center for Psychosocial Medicine, University Hospital Heidelberg, Heidelberg, Germany; c Department of Psychology, Harvard University, Cambridge, MA, USA; d Department of Psychology, College of Liberal Arts, University of Minnesota, Minneapolis, MN, USA; e University of Cologne, Faculty of Medicine and University Hospital Cologne, Department of Child and Adolescent Psychiatry, Psychosomatics and Psychotherapy, Cologne, Germany; f Department of Child and Adolescent Psychiatry, Medical University Vienna, Vienna, Austria; g Department of Child and Adolescent Psychiatry and Psychotherapy, University of Ulm, Ulm, Germany; h School of Psychology, Victoria University of Wellington, Wellington, New Zealand; i Department of Psychosomatic Medicine and Psychotherapy, Central Institute of Mental Health, Medical Faculty Mannheim, Heidelberg University, Germany; j Department of Psychiatry, School of Medicine, University of Utah, Salt Lake City, UT, USA; k Department of Psychiatry and Behavioral Sciences, Medical School, University of Minnesota, Minneapolis, MN, USA

**Keywords:** Nonsuicidal self-injury, Neurobiology, Temporal framework, Traits, States

## Abstract

Nonsuicidal self-injury (NSSI) is a serious clinical problem, particularly for adolescents and young adults. NSSI is a complex behavior that emerges through the intersecting effects of social, psychological, and biological mechanisms. Although the social and psychological contributions to risk for developing NSSI are relatively well understood and have guided the development of effective psychosocial treatments for self-injury, the biological mechanisms underlying NSSI have just begun to come to light. To evaluate and categorize the biological research conducted on the topic of NSSI, we propose a model that distinguishes between trait and state markers. According to this model, risk factors and mechanisms involved in NSSI can be distinguished into both trait and state factors. We review the existing evidence on *distal biological traits* (predictors) of NSSI, *proximal biological traits* (correlates) of NSSI, and *biological states* directly preceding or following NSSI. We conclude by providing recommendations for future research on the neurobiology of NSSI.

## 1. Introduction

### 1.1. Nonsuicidal self-injury

Nonsuicidal self-injury (NSSI) is a serious clinical problem, particularly for adolescents and young adults. NSSI is defined as “the deliberate, self-inflicted damage of body tissue without suicidal intent and for purposes not socially or culturally sanctioned” (International Society for the Study of Self-Injury, ISSS), and typically manifests as cutting, scratching, or banging the body (Whitlock et al., 2006). According to a meta-analysis, the prevalence estimate for a single episode of NSSI in non-clinical samples is 17.2 % among adolescents, 13.4 % among young adults, and 5.5 % among adults (Swannell et al., 2014). NSSI has been introduced as a potential discrete diagnostic entity in the 5th edition of the Diagnostic and Statistical Manual of Mental Disorders (DSM-5) in Section 3 as a disorder warranting further research (APA, 2013). The prevalence of NSSI disorder (according to DSM-5 diagnostic criteria) is estimated at 0.3–5 % among non-clinical samples of adolescents, and between 50–80 % among inpatient samples (Zetterqvist, 2015; Plener et al., 2016). Critically, NSSI is a significant predictor of subsequent suicide (Mars et al., 2019). Given its prevalence and increased mortality, the World Health Organization has recognized NSSI as one of the top five major health threats to adolescents (WHO, 2014).

### 1.2. The importance of advancing our understanding of the biology underlying NSSI

NSSI is a complex behavior that emerges through the intersecting effects of social, psychological, and biological mechanisms. Although the social and psychological contributions to risk for developing NSSI are relatively well understood and have guided the development of effective psychosocial treatments for self-injury (Kothgassner et al., 2020), the biological mechanisms underlying NSSI have just begun to come to light. To date, no somatic treatments such as psychopharmacology or neuromodulation are available for treating NSSI (Plener et al., 2018). Addressing this gap first requires greater knowledge of the biological mechanisms, which underlie dispositional factors, as well as the onset and maintenance of NSSI.

Critically, NSSI is a behavior that emerges during early adolescence (Plener et al., 2015), a time period notable for significant brain development (Giedd et al., 1999, 2009). The neuroplasticity inherent to such developmental periods represents both a vulnerability for the onset of mental health problems during adolescence, as well as an opportunity for potentially enhanced intervention due to increased malleability (Paus et al., 2008). Advancing our understanding of how and when neurophysiological processes go awry in adolescents who engage in NSSI will be critical in developing optimally-timed interventions designed to address the particular profile of interacting neurobiological, psychological and social mechanisms that are perpetuating a given individual’s NSSI.

Here we provide a review of recent neurobiological research on NSSI and propose a theoretical model for understanding recent findings and guiding future inquiries. Given the complexity of the brain and its manifold connections, numerous research approaches are required to begin understanding the multiple intersecting systems that are implicated in the onset and maintenance of NSSI during adolescent development. The research approaches reviewed here include: (a) genetics and epigenetics, (b) brain structure, connectivity, activation patterns, neurotransmitters, and behavioral indicators of brain functioning (neurocognitive functioning), and (c) peripheral physiology including the autonomic nervous (ANS) and the neuroendocrine systems. These research approaches have been applied and integrated to examine multiple biological systems implicated in NSSI including those represented in negative affect, stress, pain, and social functioning. Critically, identified disruptions in these biological systems may reveal targets for developing novel interventions (e.g., psychosocial, pharmacology, neuromodulation).

### 1.3. Model of distal and proximal trait biology as well as biological states around NSSI

To evaluate and categorize the biological research conducted on the topic of NSSI, we propose a model that distinguishes between trait and state markers, and thus is in line with advanced biological models of other mental disorders (e.g., schizophrenia; Chen et al., 2006). According to such a model, risk factors and mechanisms involved in NSSI can be distinguished into both trait and state factors.

**Fig. 1.**
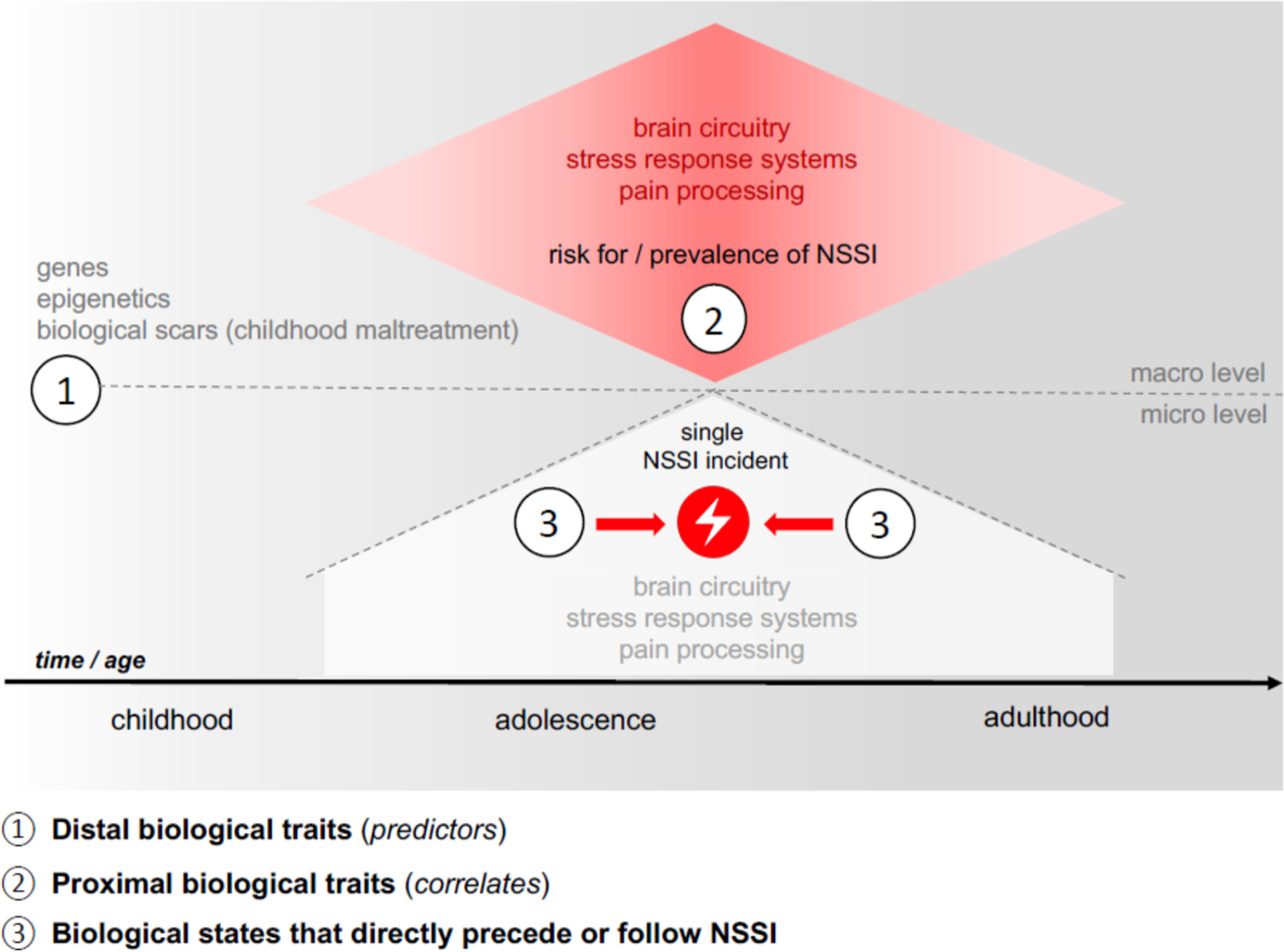
Model of different categories of the biological factors in NSSI.

A **trait** is a relatively stable, enduring characteristic or pattern of behavior or biological functioning, which plays an antecedent, possibly causal, role in the pathophysiology of psychiatric disorders. This broader definition of traits includes the manifestation of altered behavioral and biological processes that are not necessarily linked to NSSI, but to functional abnormalities related to the predisposition of this behavior. Some traits may serve as **distal** risk factors, meaning that a biological predisposition or vulnerability for NSSI was already present at birth or early in life, or developed over a long period of time (so-called distal biological traits; see Fig. 1).

However, most biological traits that have been investigated in the context of NSSI are more **proximal** in nature, as studies have predominantly used cross-sectional designs, which capture more proximal correlates of ongoing NSSI (so-called proximal biological traits; see Fig. 1). These traits represent potentially underlying biological processes that are of moderate stability but are not expected to change within days or even weeks. In contrast, a **state** is a temporary way of being (i.e., thinking, feeling, behaving, and relating) or a transient biological marker or mechanism that reflects the current status of clinical manifestations in individuals with NSSI. Research on biological states in NSSI has been particularly focused on proxies of NSSI incidents (i.e. the experimental exposure to stress or pain), trying to understand the biological processes that unfold across an NSSI episode (i.e., the moments immediately before, during, and immediately after; see Fig. 1). According to our model, the biological characteristics related to the development, onset, execution, and maintenance of NSSI can be divided into:

- distal biological traits (i.e., genetic factors or risk factors that predict the development of NSSI),
- proximal biological traits defined as underlying biological alterations observed among individuals who engage in NSSI (i.e., alterations in brain structure and functioning as well as endocrine and physiological systems), and
- biological states that directly precede or follow NSSI (i.e., changes in brain functioning as well as reactivity of endocrine and physiological systems).

We aim to summarize the existing evidence on the biology of NSSI following this classification approach. As a result, specific biological systems are discussed in multiple sections within this review depending on their potential fit into the model described above.

## 2. Distal biological traits (predictors)

Although we are still in the early stages of identifying and understanding the distal risk factors present in childhood that increase an individual’s vulnerability to NSSI, some research to date has focused on genetic predisposition, childhood maltreatment, and the interaction between genes and maltreatment.

### 2.1. Genes

Currently, little is known about potential genetic influences on the development of NSSI, and the few studies that have investigated the role of genes have yielded mixed findings. In a twins study, genetic influences explained only thoughts about NSSI, but not NSSI behavior (Jang et al., 1996). In contrast, based on a large sample of adult twins, genetic factors explained about 37 % and 59 % of variance in NSSI among males and females, respectively (Maciejewski et al., 2014). A small body of research has investigated the role of specific genes in the manifestation of NSSI, with a focus on genes relevant to neural systems implicated in emotion. First, several studies have targeted a polymorphism of the serotonin-transporter linked promoter region (5-HTTLPR). No significant main effects of the 5-HTTLPR on the occurrence of NSSI were found in a sample of male prisoners (Gorodetsky et al., 2016) or between groups of adults receiving inpatient care who reported a history of suicide attempts or NSSI (Dell’osso et al., 2013). Steiger et al. (2011) failed to find a relationship between variants of the 5-HTTLPR and self-harming behavior, defined as NSSI and/or suicidal behavior, in independent samples of adult females with eating disorders. However, in the same study, high-function variants of the monoamine oxidase A (MAOA) gene were associated with increased risk of self-harming behavior (Steiger et al., 2011). With regard to polymorphisms of the catechol-O-methyl transferase (COMT) gene, no significant differences were found between groups of adults receiving inpatient care who reported a history of suicide attempts or engagement in NSSI (Dell’osso et al., 2013). In contrast, Bernegger et al. (2018) found a haplotype of COMT polymorphisms (consisting of rs737865, rs6269, and rs4633) was associated with NSSI among adults with affective disorders. A main effect on NSSI was further reported for a polymorphism of the gene encoding for G protein β3 (GNβ3) among people from different age groups with depressive disorders (Joyce et al., 2006).

To date, only three studies have focused on potential gene-environment interactions in predicting NSSI. Hankin et al. (2015) reported results from two independent population-based samples of children and adolescents, showing that youth with at least one short variant of the 5-HTTLPR exhibited higher rates of engagement in NSSI when experiencing severe interpersonal peer stress. Moreover, a relationship between reports of an invalidating emotional environment during childhood and the development of NSSI was found only among Val carriers of the brain derived neurotrophic factor (BDNF; Val66Met polymorphism) compared to Met carriers (Hankin et al., 2015). In a population-based sample of Chinese adolescent males, Gao et al. (2021) found a three-wave interaction between experiences of childhood abuse with variants of the MAOA and COMT genes in the prediction of NSSI: only in a subgroup of carriers with the T allele of the MAOA gene and the Met allele of COMT, there was no significant relation between experiences of abuse and the development of NSSI.

A significant limitation of most of the research to date reporting genetic main or interaction effects on NSSI is that these studies have relied on sample sizes of approximately 50–500 individuals. For this reason, such studies have limited potential to detect the usually small effects of single gene variants and may be strongly influenced by sampling errors. To our knowledge, only one study relied on a large population-based sample of close to 100,000 adults; in this genome-wide association study, no polymorphism was significantly related to NSSI (Strawbridge et al., 2019).

### 2.2. Epigenetics

To the best of our knowledge, only one study has thus far investigated the role of epigenetic changes in the context of NSSI. Martín-Blanco et al. (2014) found that methylation status of the promoter region of the glucocorticoid receptor gene (NR3C1, exon 1 F) was positively associated both with a history of childhood maltreatment and with severity of self-injurious behaviors in a sample of adults with borderline personality disorder (BPD). While the extant evidence suggests that childhood adversity may cause epigenetic alterations that increase vulnerability for NSSI, there are no studies to date investigating any biological markers that precede the onset of NSSI, neither in optimal rearing environments nor in the context of stress or childhood adversity.

### 2.3. Biological manifestations of childhood maltreatment

A large body of literature has reported associations between the development of NSSI and a history of adverse childhood experiences, including child abuse and neglect (Serafini et al., 2017) and other forms of childhood adversity (Kaess et al., 2013). Building on data from a large study of families (Paul and Ortin, 2019) as well as on a representative community population sample (Brown et al., 2018), it is still a matter of continuing debate, whether a history of child abuse and neglect is best understood as a direct risk factor for NSSI, or rather as a factor creating a broader, more indirect vulnerability for psychiatric sequelae, which then increases the likelihood of engaging in NSSI. Regardless, it is important to consider neurobiological findings from studies of child abuse and neglect as they may shed light on later vulnerability to NSSI and can foster our understanding of how environmental factors can influence biological systems. Although an in-depth discussion of the mechanisms involved in child abuse and neglect is beyond the scope of this review, a large body of literature, which has been summarized in various reviews, has described how chronic child abuse and neglect leads to alterations in the hypothalamic-pituitary-adrenal (HPA) axis (Kuhlman et al., 2017), brain structure and function (Teicher et al., 2016), and inflammation (Danese and Baldwin, 2017). Taken together, this research suggests that these biological systems may be particularly important to consider in relation to NSSI.

## 3. Proximal biological traits (correlates)

Most neurobiological research on NSSI to date has consisted of cross-sectional studies comparing individuals with versus without NSSI. As such, this body of research has provided new knowledge about proximal biological traits associated with NSSI. As reviewed below, neurobiological domains of focus have included brain circuitry, peripheral stress response systems including the autonomic nervous system and the HPA axis, and pain systems. In this section we discuss the research examining these systems and their function in general, while in Section 3 (“Biological States that Directly Precede or Follow NSSI”) we discuss current knowledge about how these neurobiological systems function surrounding NSSI episodes.

### 3.1. Brain circuitry correlates of NSSI

Measurement of brain structure and function using non-invasive neuroimaging techniques is a key strategy to identifying proximal biological traits associated with NSSI. Although it is important to note that neuroimaging research on NSSI is still in the early stages, here we integrate the extant literature in terms of systems of neuro-behavioral functioning.

#### 3.1.1. Circuits underlying emotion expression and regulation

Since NSSI is most often (but not exclusively) used to regulate emotions (Nock, 2009; Plener et al., 2016), research investigating the processes involved in emotion experience and regulation is important for shedding light on this behavior. Studies on individuals with NSSI commonly reveal deficits in cognitive emotion regulation skills such as compromised reappraisal and a tendency to suppress emotions (Andover and Morris, 2014; Tatnell et al., 2017). To date, neuroimaging studies have provided converging evidence implicating alterations in fronto-limbic neural systems in NSSI, which are centrally involved in emotion processing and expression (Phillips et al., 2003). Key fronto-limbic structures include the amygdala, a core region for the processing of negative emotion and threat (Ledoux, 2000; Lindquist et al., 2016); frontal regulatory regions such as the anterior cingulate cortex (ACC), medial prefrontal cortex (PFC) and dorsolateral PFC; the hippocampus (emotional memory and HPA regulation; (Lupien et al., 2009); and the insula (emotional salience processing; (Seeley et al., 2007). Structurally, NSSI has been associated with reduced grey matter volume in the insula and the ACC in adolescent outpatients (Ando et al., 2018). An initial diffusion imaging study reported that adult outpatients with BPD and self-injurious behavior showed compromised white matter microstructure within the frontal lobe (Grant et al., 2007). This was further supported by a study that found that adolescents and young adults with NSSI showed lower white matter integrity compared to healthy controls in widespread circuits, and that reduced white matter integrity of fronto-limbic tracts correlated with NSSI duration (Westlund Schreiner et al., 2019). Functionally, adolescents with NSSI showed greater resting-state functional connectivity between the amygdala and posterior regions of the ACC relative to healthy controls (Westlund Schreiner et al., 2017).

Functional imaging paradigms probing brain activation in the context of emotional experiences provide further support implicating emotion reactivity and regulation in NSSI. In the first fMRI study to examine this, adolescent patients with NSSI showed greater amygdala and ACC activation in response to negative, positive and neutral pictures compared to healthy controls (Plener et al., 2012), suggesting heightened emotional reactivity in this population. Notably, in a study with non-hospitalized adults with NSSI (but without BPD), these effects were not apparent in comparison to a clinical control group without NSSI matched for psychopathology, although the NSSI group was still less able to down-regulate their amygdala activity using reappraisal than matched controls (Davis et al., 2014). Taken together, research findings to date implicate fronto-limbic circuitry in neurobiological vulnerability to impaired processing of emotional stimuli in adolescents with NSSI.

#### 3.1.2. Social / self neural systems

The neural systems that underlie how young people think about themselves are highly relevant to understanding NSSI. The stressful events that trigger NSSI episodes are often social in nature. People who self-injure are more likely to have problematic relationships with their parents and are often victims of bullying (Esposito et al., 2019; Liu et al., 2018; Tatnell et al., 2017). In addition, interpersonal stressors such as trouble with parents or a serious argument with a close friend predict the first onset of self-injury (Kaess et al., 2020). The brain networks underlying processing of self and others overlap to some extent with the front-limbic networks discussed above; the “social brain” is comprised of medial PFC, temporoparietal junction, posterior superior temporal sulcus, inferior frontal gyrus, interparietal sulcus, amygdala, ACC, and anterior insula (Blakemore, 2008) while brain networks specific to self processing is thought to be comprised of medial cortical regions (medial PFC/ACC, ventrolateral PFC, posterior cingulate and precuneus) together with angular gyrus, temporo-parietal junction, temporal pole, posterior temporal sulcus (Pfeifer and Peake, 2012). Together, many of these regions also contribute to the salience and default mode networks. A recent study found that in a sample of adolescents, patients with depression and NSSI demonstrated reduced connectivity at rest within the salience and default mode networks relative to patients with depression but no NSSI and healthy controls (Ho et al., 2021). A strength of this study is the use of both clinical and healthy controls, which suggests that findings may be unique to NSSI rather than due to general psychopathology.

Using social exclusion paradigms, several studies have now demonstrated that compared to healthy control participants, adolescent and adult patients with NSSI demonstrate greater activation of fronto-limbic / social brain network regions during social exclusion including the ACC (Brown et al., 2017; Malejko et al., 2019), insula (Malejko et al., 2019), ventrolateral and medial PFC, and parahippocampal gyrus (Groschwitz et al., 2016). These findings of fronto-limbic overactivation to social threat (along with elevations on self-report measures of rejection sensitivity) have generally been interpreted as reflecting higher rejection sensitivity coupled with unsuccessful efforts to regulate feelings of rejection among those with NSSI. In a study where adolescents took either their own or someone else’s perspective to judge self-related characteristics, adolescents with both depression and NSSI had higher limbic activity across all perspective-taking conditions, compared to both depression-only and healthy controls, especially when taking their mother’s perspective, and particularly for those who perceived low emotional support from their mothers (Quevedo et al., 2016). Hence, the neural circuits underlying emotion processing intersect with those underlying processing of self / other, likely reflecting the emotional difficulties that occur in social contexts given that interpersonal difficulties are common for individuals with NSSI.

#### 3.1.3. Cognitive control

Research has provided some some evidence that individuals with NSSI may demonstrate greater impulsivity, which could help explalin their vulnerability in developing and maintaining this behavior. The deliberate implementation of emotion regulation strategies is a complex process which entails both a decision *if* and *how* to regulate (Sheppes et al., 2014) as well as the suppression of alternative impulses. The brain regions which together execute cognitive control functions (a set of higher cognitive tasks including impulse control) include the dorsolateral PFC, ACC, dorsal parietal cortex and precentral gyrus (Williams, 2017). Some studies but not others have found correlates between impulsivity and other indexes of cognitive control with NSSI (McHugh et al., 2019). To date, brain correlates of cognitive control are a relatively understudied area in NSSI research. One study showed that during a cognitive interference task, adults with NSSI showed no deficits in suppressing distracting information on a behavioral level but showed greater dorsolateral PFC activity relative to controls. In the NSSI group, this activity was positively associated with self-reported global levels of emotional reactivity and impulsivity (Dahlgren et al., 2018). At this time, there is not yet adequate information in the neuroimaging research showing a robust pattern with regard to cognitive control systems in NSSI.

#### 3.1.4. Reward

The reward system represents an area of interest for NSSI research given open questions as to whether NSSI is maintained through either positive or negative reinforcement, and whether individuals with NSSI may exhibit reduced reward responsiveness to positive inputs or experiences. Central components of the reward network include the ventral striatum, orbitofrontal cortex (OFC) and ACC (Williams, 2017). To date, research on NSSI and reward has been mixed. While one study demonstrated *enhanced* striatal activation to monetary rewards in adolescents with NSSI (Poon et al., 2019), another study showed *lower* activity of striatum, OFC, and amygdala in adolescents with NSSI during reward processing (Sauder et al., 2016). Notably, the value of rewards is a central moderator in models on reward processing in adolescence (Galvan et al., 2006; Vaidya et al., 2013). A relatively large study reported higher OFC activity in adult patients with BPD with versus without NSSI, but only when rewards were unexpectedly high (Vega et al., 2018). Moreover, in a community sample of adults with depression, abnormal connectivity between striatal and motor/sensory regions was correlated with self-injury, but not with depression or suicidal ideation (Marchand et al., 2012) further implicating regions associated with reward processing in NSSI. Behavioral indicators of decision making under reward conditions have not consistently been linked with NSSI (McHugh et al., 2019). Taken together, the findings to date on the reward system in NSSI are mixed, which will require further studies to disentangle.

In sum, neuroimaging studies have begun to investigate the role of neural systems involved in processing emotion, thoughts about self and other, cognitive control, and reward in NSSI. Notably, some of the neuroimaging correlates noted above have also been associated with stress exposure, especially during early life years (Lupien et al., 2009), suggesting a potential connection between more distal risk factors and proximal NSSI biomarkers. Given that these neurobiological systems are still undergoing development during adolescence, a critical next step in research will be to better understand how abnormalities in these systems change over time across development and in the context of treatment interventions.

### 3.2. Peripheral stress response systems

Biological systems centrally implicated in the human stress response potentially contribute to the risk for developing and maintaining NSSI. The autonomic nervous system (ANS) and the HPA axis are considered the two most important stress response systems, allowing the organism to functionally adapt to changing environmental demands. Given that individuals often engage in NSSI to regulate unpleasant experiences (Edmondson et al., 2016; Klonsky and Glenn, 2009), and that interpersonal stressors have particular influence for those individuals (Kaess et al., 2020), recent research aimed to answer the question whether the physiological stress response systems may be altered in NSSI, including examination of both the basal functioning and the reactivity of these systems to acute stressors among individuals with NSSI.

#### 3.2.1. Autonomic nervous system

The ANS regulates organ function in the context of rest (parasympathetic branch) and stress (sympathetic branch). Predominantly, ANS activity in NSSI is studied by investigating its innervation of the heart and skin. Psychophysiological methods allow the quantification of resting ANS activity and phasic response to certain stimuli. Initial evidence for potential alterations in resting ANS functioning came from comparisons of vagally-mediated heart rate variability (HRV)—a proxy of cardiac parasympathetic activity—among adolescents with deliberate self-harm and age-matched controls (Crowell et al., 2005). Results indicated that adolescents with deliberate self-harm demonstrated reduced vagally-mediated HRV (i.e., lower parasympathetic activity) at rest. In contrast, resting cardiac function, as indexed by heart rate (mixed sympathetic and parasympathetic influence) and vagally-mediated HRV was not altered in a study comparing outpatient adolescents with NSSI to healthy controls, although cardiac function was negatively related to symptoms of BPD (Koenig et al., 2017b). Greater severity of BPD pathology was also associated with increased heart rate and reduced HRV and these findings were recently replicated in an independent sample of adolescent patients (Weise et al., 2020). Additional analyses of Koenig et al. (2017b) revealed that the relationship between resting cardiac function and dimensional personality pathology also held longitudinally (Koenig et al., 2018). Overall, these data suggest that individuals with NSSI are characterized by an ANS profile reflecting greater sympathetic dominance, as a function of underlying dimensional personality pathology (e.g., emotion regulation deficits).

In terms of cardiac reactivity to acute stress, adolescents with deliberate self-harm showed greater vagally-mediated heart rate variability during a sadness induction than age-matched controls (Crowell et al., 2005), but adolescents with NSSI showed no differences in heart rate during a standardized psychosocial stress procedure (Kaess et al., 2012). Adults with eating disorders and NSSI also demonstrated no differences in in-task heart rate variability (during biofeedback video game play) compared to both healthy controls and adults with eating disorders and no NSSI (Giner-Bartolome et al., 2017).

Moving beyond cardiac indicators, evidence for systematic differences in NSSI across other domains of stress reactivity is mixed. Changes in skin conductance partially reflect the influence of the sympathetic nervous system on eccrine sweat gland secretion and provide a measure of physiological arousal. Nock and Mendes (2008) compared skin conduction of a large sample of adolescents and young adults with NSSI to matched controls during a frustration task, finding that the NSSI group demonstrated greater physiological reactivity than controls. Notably, this difference was most pronounced at the end of the 14-minute task. In contrast, other research has found no difference in skin conductance levels by self-injury status during a standardized psychosocial stress procedure (Tatnell et al., 2018), or in skin conductance responses during a sadness induction (Crowell et al., 2005).

#### 3.2.2. Hypothalamic–Pituitary–Adrenal Axis

The HPA axis consists of the reciprocal neuroendocrine action of the hypothalamus, the anterior pituitary gland, and the adrenal glands and constitutes a major stress response system. Given that states of heightened stress typically precede acts of NSSI, examination of the functioning of this dynamic system under resting conditions, across the diurnal cycle, and in response to acute stressors has been critical to advancing our understanding of NSSI. Although studies applying ecologically valid designs (e.g., measuring ANS or HPA activity and reactivity surrounding events of NSSI in everyday life) are currently limited, lab-based experiments (e.g., stress induction manipulations) may help to understand differences in ANS and HPA stress-reactivity in patients engaging in NSSI. Initial research found that adolescent girls with NSSI showed an attenuated salivary cortisol response to a standardized psychosocial stress procedure compared to those without a history of NSSI, suggesting a blunted cortisol response in NSSI (Kaess et al., 2012). Plener et al. (2017) found that adolescent girls from a longitudinal sample of children growing up in precarious living conditions, with a lifetime history of deliberate self-harm showed lower plasma cortisol over the course of the same psychosocial stress procedure than did those without deliberate self-harm. However, in contrast to Kaess et al. (2012), the self-harm and control groups showed a similar reactivity pattern over the course of the stress procedure, perhaps reflecting differences in cortisol assessment (blood plasma compared to saliva). More recently, Klimes-Dougan et al. (2018) replicated the blunted salivary cortisol response in a larger sample of adolescents with both depression and NSSI compared to healthy controls and adolescents with depression but no NSSI. While this pattern of aberrant HPA axis functioning was present in both males and females, the number of male participants was low, leaving some uncertainty as to whether males with NSSI may exhibit a different pattern (Klimes-Dougan et al., 2018). Notably, the severity of self-harm moderated the extent of blunted cortisol: adolescents with recurrent NSSI had lower levels of cortisol than those with one or two lifetime episodes of NSSI.

In addition to more standard reactivity paradigms, alternative methods of measuring HPA may provide a more nuanced picture of HPA functioning. Reichl et al. (2016) found that compared to matched healthy controls, adolescents who had engaged in five or more episodes of NSSI demonstrated a greater cortisol awakening response (sharp increases in cortisol in the first 30–45 min after awakening), perhaps indicating greater expectation of difficulty during the day (Kramer et al., 2019), or poorer sleep quality. Notably, this elevated cortisol was not maintained throughout the day, as the NSSI and healthy control groups demonstrated similar diurnal slopes as well as basal cortisol levels across the past three months (as indexed by hair cortisol levels). In addition, childhood adversity moderated the diurnal cortisol slopes. Healthy controls who had experienced childhood adversity had flatted diurnal cortisol slopes while adolescents with NSSI who had experienced adversity had steeper slopes. Beauchaine et al. (2015) assessed alterations in resting HPA axis functioning among adolescent girls with depression only compared to adolescent girls with depression and self-harm using a dexamethasone suppression test (DST). Consistent with the interpretation that NSSI may be characterized by maladaptive neuroendocrine processes, lower post-DST cortisol levels were associated with self-harm status over and above reports of internalizing and externalizing behavior. Most recently, in a study of adolescents with NSSI and their siblings, hair cortisol and saliva cortisol were measured before and after diagnostic interviews (Reichl et al., 2019). Although the NSSI group reported higher levels of childhood adversity and showed higher levels of hair cortisol, they presented with a decrease of salivary cortisol during the retrieval of childhood adversities as part of the diagnostic interview (Reichl et al., 2019), while no such changes were apparent in the sibling group. These findings point to an alteration in the HPA axis due to child abuse and neglect, which acts as a (often chronic) stressor with the potential to attenuate the cortisol response.

Importantly, a developmental progression from childhood to adolescence may take place in which adverse experiences may be related to a shift in HPA axis functioning. For example, a longitudinal study tracking young women from age 6 to age 30 found that females with a history of abuse who initially had high cortisol later showed low cortisol levels as adults (Trickett et al., 2010). Results from another longitudinal study found a relationship between childhood adversity and larger pituitary volumes leading to later HPA axis attenuation (Kaess et al., 2018), thereby supporting the hypothesis that childhood adversity may contribute to blunted cortisol response in adolescence and adulthood. However, long-term longitudinal research on HPA functioning to understand potential HPA axis functioning shifts in adolescents with NSSI has yet to be conducted.

### 3.3. Pain systems

Since NSSI by definition causes tissue damage, which typically involves some degree of pain, pain sensitivity has been an active area of NSSI research. The central question in this field of research is whether reduced pain sensitivity represents a proximal trait, increasing the risk to engage in NSSI in the future, or rather is a consequence of previous NSSI (i.e., adaptation hypothesis). Many studies in this area have been conducted with adults with a BPD diagnosis (e.g., Schmahl et al., 2006), while others have focused more specifically on adults with NSSI in community samples (e.g., Hooley et al., 2010). Comparison samples unusually involve healthy controls. In addition, various types of laboratory pain stimuli have been used, including thermal (Franklin et al., 2012; Koenig et al., 2017a,c) electrical (Weinberg and Klonsky, 2012), and mechanical pressure (McCoy et al., 2010; Glenn et al., 2014) pain stimuli. Recent technological advances introduced a blade stimulus to induce non-injurious sharp mechanical pain (Shabes et al., 2016), that is considered to better mirror the pain experience during actual acts of NSSI (i.e., cutting the skin). Although there is ongoing debate concerning the ecological validity of laboratory-based pain induction in people with NSSI, findings on reduced pain sensitivity in those engaging in NSSI are well-replicated.

A review and meta-analysis of data from 32 different studies, including various samples, concluded that individuals engaging in NSSI report greater pain threshold, greater pain tolerance and lower self-reported pain intensity compared to healthy controls (Koenig et al., 2016). Adults and adolescents with NSSI also show elevated pain endurance—a measure that reflects willingness or ability to endure pain after the onset of pain (pain tolerance minus pain threshold (see Glenn et al., 2014; Hooley et al., 2010). Cross-sectional findings in adults with BPD further suggest that pain sensitivity may normalize following cessation of NSSI (Bekrater-Bodmann et al., 2015; Ludäscher et al., 2009). However, the only longitudinal study on pain sensitivity in adolescent NSSI showed that greater reduction in NSSI frequency after one-year was associated with increased pain tolerance. This finding suggests that individuals may disengage from NSSI once pain tolerance gets too high—perhaps due to attenuated effectiveness of NSSI for regulating emotion (Koenig et al., 2017a). Although it is currently not possible to draw conclusions concerning the causal relationship between pain sensitivity and NSSI, the later finding supports the idea that pain sensitivity presents a proximal trait for the engagement in NSSI and the termination of the behavior. How age-related developmental changes may impact the association between pain sensitivity and NSSI warrants further research.

Pain sensitivity is a complex construct, driven by psychological and biological mechanisms. On the biological level, findings of diminished pain perception in people who engage in NSSI have given rise to suggestions of endogenous opioid involvement. Four distinct classes of endogenous opioids can be distinguished: endorphins, enkephalins, dynorphins, and endomorphins (see Bresin and Gordon, 2013). In a pioneering study, Coid et al. (1983) noted that plasma levels of enkephalins (but not β-endorphin) were *higher* in adults with BPD who engaged in NSSI (*n* = 10) compared to healthy controls. However, more recent research in larger samples suggests that levels of endogenous opioids may be *lower* in people who engage in NSSI compared to controls. Stanley et al. (2010) examined CSF levels of β-endorphin, met-enkaphalin, and dynorphin in adults with depression, a cluster B personality disorder (primarily BPD) and who had a history of suicide attempts; half of the sample had a history of NSSI. β-endorphin and met-enkephalin levels were significantly lower in those with versus those without NSSI. No significant differences were noted for dynorphin. Based on these findings, Stanley et al. (2010) proposed an opioid deficiency model, with NSSI being used in an effort to restore homeostasis by increasing endogenous opioids. Findings suggesting a reduced resting plasma β-endorphin levels have recently been replicated in adolescents engaging in NSSI (van der Venne et al., 2020). However, whether an opioid deficiency predates NSSI or is a consequence of NSSI engagement remains unclear. Further, studies were not able to confirm the suggested link between endogenous opioids and altered pain sensitivity in adolescents with NSSI (van der Venne et al., 2020).

It remains difficult to reconcile how lower resting levels of endogenous opioids are consistent with generally lower pain sensitivity in people who engage in NSSI. Increased activity at mu- and delta receptor sites is, in principle, associated with a decrease in pain sensitivity, consistent with the use of morphine (a mu-receptor agonist) being used clinically for pain management. If resting levels of β-endorphin and metenkephakin are low, activity at mu and delta receptor sites would be expected to be reduced, leading to predictions of *increased* pain perception sensitivity. Of course, if an episode of NSSI is currently in process or has recently occurred, this would be expected to increase β-endorphin levels, providing an explanation for *reduced* pain sensitivity (i.e., increased pain thresholds and longer pain endurance). However, this is not the case in most instances. An alternative explanation for pain analgesia (i.e., lower pain sensitivity) in people who engage in NSSI may be that the pain of an experimental stimulus (e.g., heat, cold, pressure pain) triggers the release of β-endorphin. If we further assume that low resting levels of endogenous opioids result in mu and delta receptors becoming highly sensitive to opioids, this could potentially create a context in which pain analgesia might be reported. Empirical tests of this hypothesis remain to be conducted. Initial evidence from an ecological momentary assessment (EMA) study points to low salivary β-endorphin levels, which are increased by NSSI events (Störkel et al., 2020).

Efforts to examine the role of these neuropeptides in NSSI are hampered by the overall limitation that neuropeptide levels obtained from plasma may not reflect neuropeptide levels centrally (De Riu et al., 1997). Also problematic for the opioid deficiency model is that NSSI tends to occur when a person is in a state of stress (Armey et al., 2011). Yet stress is associated with increased opioid activity (Butler and Finn, 2009). This means that: (a) NSSI is more likely to happen under conditions of elevated rather than reduced levels of endogenous opioids, and (b) opioids are not linked only to positive affective states. This pattern is the inverse of the opioid homeostasis model and challenges the idea that NSSI functions to increase low levels of endogenous opioids. However, the link between stress and increased opioid activity provides a possible explanation for the reduced pain perception that is found in many people who engage in NSSI. In summary, based on current knowledge, although it is certainly reasonable to expect that endogenous opioids might be involved in the mechanisms underlying reduced pain sensitivity in NSSI and NSSI per se, it is difficult to integrate these findings into a coherent theoretical framework at the present time. Multimodal studies assessing pain sensitivity, opioid levels at rest and following NSSI events in longitudinal designs are necessary to further understand these associations. While opioid blockers do reduce NSSI engagement to some extent in people with developmental disabilities (Symons et al., 2004), treatments that increase opioid levels have also been shown to reduce NSSI (Nixon et al., 2003). Thus, although reduced pain sensitivity in NSSI has been well replicated, models of NSSI that are primarily based on imbalances of endogenous opioids are somewhat premature, given the current state of the evidence in this area.

## 4. Biological states that directly precede or follow NSSI

Although we do not yet have the availability of ethical, practical research approaches for measuring biological states directly before or during real-time NSSI events, as a surrogate approach, researchers have developed experimental paradigms to simulate NSSI to allow examination of biological states during proxy NSSI experiences. Such paradigms include application of painful stimuli such as heat, and even induction of injury using a blade. Application of such paradigms to measure self-reported stress and arousal has thus far suggested that the reduction of stress levels after a pain stimulus occurs regardless of whether the pain stimulus is associated with tissue injury (Willis et al., 2017), and that seeing blood appears to have an additional stress-dampening effect (Naoum et al., 2016). Studies applying these paradigms to examine evolving biological states associated with NSSI episodes, reviewed below, include approaches using neuroimaging to examine brain circuitry and stress-response paradigms to study peripheral response systems in the context of NSSI-like paradigms.

### 4.1. Brain circuitry changes during experimental NSSI paradigms

Studies to date (primarily focusing on adults with BPD and NSSI) that have applied neuroimaging approaches with NSSI-simulating experimental paradigms have generally implicated fronto-limbic (emotion expression/regulation) systems and pain systems in the neurobiological state changes that occur in the moments before, during and after NSSI episodes. In a first study by Schmahl et al. (2006), adult inpatients with BPD and NSSI and healthy controls underwent an fMRI scan while heat stimuli were applied to their hands. The BPD group showed greater pain thresholds than healthy controls. When considering the effects of pain, individually-adjusted to threshold, the BPD group showed lower activation in amygdala and ACC but greater activation in dorsolateral PFC than healthy controls, suggesting a mechanism in which top-down-regulation of emotional components of pain modulate affective appraisal of pain. Kraus et al. (2010) explored neural processing of NSSI behavior in adult inpatients with and without BPD by measuring neural activation while participants imagined different aspects of an NSSI episode (e.g., the situation triggering an NSSI episode, emotional and cognitive reactions to the triggering situation, the NSSI act, the feelings after the NSSI act (Kraus et al., 2010). During the description of the trigger situation, people with BPD showed lower activation in the OFC but higher activation of the dorsolateral PFC than the control group. While imagining the NSSI act, the BPD group showed a significant decline in mid-cingulate activity (Kraus et al., 2010). To further examine the interaction between self-inflicted pain and affect regulation, Niedtfeld et al. (2010) used picture stimuli to elicit negative affect and thermal stimuli to induce heat pain in adults with and without BPD. While negative pictures were associated with greater fronto-limbic (amygdala, ACC, insula) activation in BPD versus controls, there was some evidence for attenuated activation in limbic areas (amygdala, insula) in response to painful stimulation for both healthy and BPD individuals (Niedtfeld et al., 2010). In a re-analysis of this data to further probe brain mechanisms underlying limbic deactivation (Niedtfeld et al., 2012), researchers applied psychophysiological interaction analyses to assess how functional connectivity of amygdala, insula, and ACC changes across negative emotion and pain contexts. These results showed that painful sensory stimuli, as opposed to warmth perception, resulted in enhanced negative fronto-limbic coupling in patients BPD, suggesting a mechanism of inhibition of limbic arousal (Niedtfeld et al., 2012). A study that used an incision paradigm demonstrated a decrease of amygdala activity after incision and an additional restitution of post-stress amygdala-mPFC coupling following incision in an adult community sample with BPD and NSSI (Reitz et al., 2015). The differential effect of incision versus a control condition was only found immediately after the painful stimulation (in the first five minutes), but disappeared afterwards (Reitz et al., 2015), underscoring the importance of research approaches that can capture the transience of neurobiological states that transpire across NSSI episodes. In summary, early evidence from NSSI-simulation fMRI paradigms has shed light on how fronto-limbic and somatosensory neural systems interact and change across the NSSI episode in adult populations showing a high frequency of NSSI.

### 4.2. Peripheral stress response systems - changes across NSSI episodes

Given the role of pain in NSSI, research has also considered ANS and HPA reactivity to painful stimulation in particular. Koenig et al. (2017c) assessed how the response of adolescent girls with NSSI (*n* = 30) compared to matched controls (*n* = 30) during a cold pain stimulation. Adolescents with NSSI showed a greater cortisol response to pain than did controls, a finding inconsistent with previous research showing a blunted cortisol response to stress in NSSI (Kaess et al., 2012; Klimes-Dougan et al., 2018). Although the NSSI group showed similar systolic and diastolic blood pressure following painful stimulation, compared to matched controls, adolescents with NSSI demonstrated less vagal withdrawal (i.e., greater heart rate variability) during the anticipation of pain, as well as a slower return to baseline (i.e., decreased heart rate variability) during recovery from pain. The relative delay in recovery was associated with greater self-reports of better body awareness in those engaging in NSSI, an interesting finding suggesting a biological basis of the anti-dissociative function of NSSI based on autonomic arousal. Additional analyses of the NSSI subsample revealed that this prolonged autonomic arousal differed as a function of childhood trauma; adolescents with NSSI who reported greater severity of childhood trauma showed greater decreases in heart rate variability both during and following painful stimulation, as well as a stronger cortisol response to pain (Rinnewitz et al., 2018). Additional evidence for arousal comes from a single case where nocturnal urinary cortisol levels were assessed for 86 consecutive nights in a woman diagnosed with BPD exhibiting pronounced self-injury (Sachsse et al., 2002). Increases in cortisol in the evenings preceding an episode of NSSI were documented, followed by a return to baseline. Another study used scalpel incisions in adult patients with BPD in comparison to healthy controls as a more valid model of NSSI and found a stress reduction in terms of both decreased heart rate and subjective arousal (Reitz et al., 2012).

Findings on HPA and ANS reactivity to stress and pain in NSSI seem conflicting at first hand. However, as detailed elsewhere (Koenig et al., 2017a,b,c), it may be the case that insufficient support by endogenous biological systems in the face of stressful situations (i.e., as indexed by cortisol hyporeactivity) may be compensated by a hyperreactivity of the respective systems towards painful stimulation. This assumption may explain maintenance of NSSI as a dysfunctional strategy to regulate stress.

## 5. Some remaining questions and future research

While not a comprehensive review, this theoretical paper identifies key insights that have been gained through neurobiological research (for a summary of key findings see Table 1) on the proximal and distal neurobiological traits, and the evolving biological states associated with NSSI, and highlights key gaps in knowledge.

One major area in need of advancement is the confirmation of findings through replication with larger samples. This is true not only for genetics research on NSSI, but also the other neurobiological approaches which have suffered from small samples that introduce the risk for spurious findings, non-representative results, and type 2 error due to low power. Along these lines, the field is still in the early stages of understanding heterogeneity in NSSI, which further adds to the need for larger samples. For example, the neurobiological mechanisms invoked in individuals who utilize NSSI for regulating negative affect likely differ from those using it to serve interpersonal functions. Future neurobiological research would benefit from study designs and sample sizes that would allow parsing out heterogeneity related to NSSI function and other sources.

A limitation of the mostly-cross-sectional neurobiological research to date has been heterogeneity across participants in NSSI severity or intractability—adolescents who have only experimented with NSSI once or twice may have very different neurobiological profiles than those with long-established patterns of frequent NSSI engagement. Given that that there is a clear developmental course of NSSI during adolescence (Plener et al., 2015), a developmental psychopathology framework might be useful to guide future studies including its clinical implications (Beauchaine et al., 2019; Cicchetti, 2014). For example, it is plausible that the biology of NSSI largely differs when comparing young self-injuring individuals (e.g. at the onset of a variety of mental disorders) with older individuals with NSSI (e.g. with long-lasting borderline personality disorder). Thus, there is a dire need for longitudinal research in order to disentangle effects of age, duration of illness and severity of illness across the life-span. In addition, prospective studies beginning with at-risk youth to track the temporal changes across development and before and after the onset of NSSI are critical. For example, although much is now known about how childhood adversity can lead to adverse biological alterations, some of which are also seen in individuals with NSSI, there are no studies to date investigating biological markers in maltreated youth which *precede* the onset of NSSI. Thus, longitudinal studies elucidating biology × environment interactions over time could shed light on important questions of risk and resilience with regards to NSSI. Other at-risk groups that would be important to study longitudinally include children with psychopathology such as depression symptoms or impulse control problems, and/or children with a familial risk for suicidal behavior.

Accumulating data show that clinically-relevant NSSI in particular is associated with high levels of psychiatric comorbidity (Ghinea et al., 2020). Thus, studying NSSI without comorbid disorders would certainly lack external validity, while also raising the concern that current empirical data on NSSI may be largely driven by underlying comorbidity. Research designs that control for underlying comorbidity may advance our understanding of specific traits and states associated with NSSI.

Although research paradigms simulating NSSI have helped advance our understanding of the biological states which evolve over the course of NSSI episodes, we still lack critical knowledge on how biological changes transpire in real-world NSSI episodes. Moving forward, studies using EMA that include biological markers (i.e., cortisol, ECG or beta-endorphin) have promise for shedding light on real-world biological states that evolve before, during and after NSSI episodes. Fifth, animal models on NSSI would allow researchers to dive deeper into the biological mechanisms of NSSI.

Animal models have made significant contributions to our understanding of basic biology, and have provided insights into a broad variety of human central nervous system pathologies (Mumtaz et al., 2018), and this includes disorders in which NSSI is manifest (Goetghebeur and Swartz, 2016). However, little has been done to directly examine the neurochemical basis of NSSI. Evidence indicates that self-injury is not unique to humans. The forms of expression of self-injury are superficially different across species, but the underlying motivations and neurochemistry may have more in common than has previously been accorded. Thus, animal models appear to be an under-utilized resource for furthering our understanding of the neurobiology of NSSI.

Finally, research using existing or novel interventions as a probe to perturb the systems under investigation holds promise for shedding further light on the biology underlying NSSI and advancing treatment development. Experimental therapeutic interventions (e.g., psychosocial interventions, cognitive training, meditation, psychopharmacology and / or neuromodulation strategies) which demonstrate target engagement (i.e., create significant change in a biological marker plausibly related both to NSSI and to the intervention) and in turn demonstrate that this biological change corresponds to clinical improvement (e.g., decrease in NSSI) hold particular promise for advancing new and improved treatments for helping individuals desist, or potentially even prevent, NSSI.

As a very final note, there is a need that future research including its presentation and dissemination sets a stronger focus on the clinical implications of the biology of NSSI. To date, the summarized findings on the biology of NSSI seem to guide and support a treatment focus on stress- and emotion regulation, and should increasingly be used to educate therapists, patients and caregivers for achieve a better understanding of the potential mechanisms for both NSSI and its treatment. However, more implications are warranted and may include a potential stratification of patients with NSSI according to their underlying biology. If we achieve a greater understanding of potential subgroups of NSSI (including their biology but also their estimated developmental course and potential response to treatment), we might one day get closer to our goal of personalized treatment for individuals with NSSI.

## Figures and Tables

**Table 1:** Summary of the key findings* on the biology of NSSI.

	Category	Key findings
Distal biological traits	Genes	Heritability of NSSI is estimated to be 40–60 %; however, no clearly identifiable genetic factors have yet been identified. A few significant gene × environment interactions have been identified suggesting that the interaction between stressors and genes may increase risk for NSSI.
	Epigenetics	There is no sufficient evidence for epigenetic alterations in the development of NSSI yet.
	Biological scares (childhood maltreatment)	Childhood adversity (and other types of acute or chronic stressors) increase risk for NSSI. Such adversity may lead to alterations in the HPA axis, brain structure and function, and inflammatory pathways, which may represent a pathway to NSSI. The latter is still to be proven.
Proximal biological traits	Brain circuitry	There is converging evidence for alterations in fronto-limbic neural systems in NSSI, which are centrally involved in emotion regulation. In the social processing systems, fronto-limbic overactivation to social threat may reflect rejection sensitivity commonly found in individuals with NSSI. While individuals with NSSI are commonly judged as “impulsive”, there is a lack of evidence for reduced cognitive control in NSSI. Similarly, research on the reward system remains inconclusive and requires further research.
	Peripheral stress response system	Individuals with NSSI seem to be characterized by an ANS profile reflecting greater sympathetic dominance. Regarding the HPA axis, there is converging evidence for an attenuated cortisol response to stress in individuals with NSSI which research on other HPA axis indices remains largely inconclusive so far.
	Pain systems	Individuals with NSSI show decreased sensitivity to pain. There is increasing evidence for a basal lack of endogenous opioids.
Biological states that directly precede or follow NSSI	Brain circuitry	There is early evidence for an interaction of somatosensory neural systems (i.e., in response to pain) and fronto-limbic systems (i. e., decrease of amygdala activity) across the NSSI episode.
	Peripheral stress response systems	There is preliminary data suggesting that NSSI (i.e., pain stimulation) may decrease subjective arousal alongside with an increase of cortisol.

* Please note that “key findings” in the table are subject of interpretation by the authors. A more details and comprehensive review of the findings can be found in the text.
